# Enhanced Moisture Stability by Butyldimethylsulfonium Cation in Perovskite Solar Cells

**DOI:** 10.1002/advs.201901840

**Published:** 2019-12-13

**Authors:** Bohyung Kim, Maengsuk Kim, Jun Hee Lee, Sang Il Seok

**Affiliations:** ^1^ Perovtronics Research Center Department of Energy Engineering School of Energy and Chemical Engineering Ulsan National Institute of Science and Technology (UNIST) 50 UNIST‐gil, Eonyang‐eup, Ulju‐gun Ulsan 44919 Republic of Korea

**Keywords:** butylammonium iodide, butyldimethylsulfonium iodide, density functional theory (DFT), humidity stability, perovskites solar cells

## Abstract

Many organic cations in halide perovskites have been studied for their application in perovskite solar cells (PSCs). Most organic cations in PSCs are based on the protic nitrogen cores, which are susceptible to deprotonation. Here, a new candidate of fully alkylated sulfonium cation (butyldimethylsulfonium; BDMS) is designed and successfully assembled into PSCs with the aim of increasing humidity stability. The BDMS‐based perovskites retain the structural and optical features of pristine perovskite, which results in the comparable photovoltaic performance. However, the fully alkylated aprotic nature of BDMS shows a much more pronounced effect on the increase in humidity stability, which emphasizes a generic electronic difference between protic ammonium and aprotic sulfonium cation. The current results would pave a new way to explore cations for the development of promising PSCs.

## Graphical abstract

The aprotic butyldimethylsulfonium‐driven MAPbI_3_ perovskite shows a much more pronounced effect on the improvement of moisture stability compared to the protic butylammonium (BA)‐based counterpart. The BA having a potential hydrogen donor, which exists on the surface and/or grain boundaries, is vulnerable to H_2_O‐induced degradation initiators, resulting in the faster hydration followed by the irreversible degradation of perovskites.

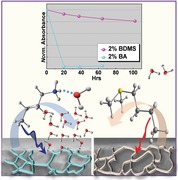

Within a few years of development, perovskite solar cells (PSCs) have achieved incredible solar‐to‐electrical power conversion efficiencies (PCEs) of over 24% under AM 1.5G.^1^ Although many PSCs have been reported, widely employed organic cations are still based on the protonated ammonium (RNH_3_^+^).^2^ Because of instability of these alkylammonium cations arising from deprotonation, stability issue poses a challenge to the commercialization of PSCs. Thus, considerable efforts are in progress to compensate for the hygroscopic nature of alkylammonium (e.g., methylammonium (MA); CH_3_NH_3_^+^) cations in perovskites.

Recently, the perovskite ((CH_3_)_3_SPbI_3_) composed of sulfonium‐based organic cations (e.g., trimethylsulfonium (TMS); (CH_3_)_3_S^+^) was synthesized and shown to exhibit high chemical stability, unlike ammonium cations. 1D perovskite was formed because of the larger size of TMS cation (**Figure** **1**), resulting in a bandgap of 3.1 eV (cf. TMS with an ionic radius of 2.44 Å vs the corresponding value of 2.17 Å for MA).^3^ Thus, tuning of the wide bandgap for low‐dimensional TMSPbI_3_ is necessary for its application to the efficient solar cells. In addition, according to our preliminary study, it was also found that the TMS is too polar to be assembled in PSCs when using typical solution processing. Nonetheless, the superior chemical stability of TMS cation attracted our attention and led us to design a new candidate to be incorporated into PSCs. This present paper reports humidity stability of PSCs assembled with butyldimethylsulfonium (BDMS) cation (Figure 1). The butylammonium (BA) cation was chosen to investigate the effect of protonated ammonium cations on humidity stability of perovskites.

**Figure 1:**
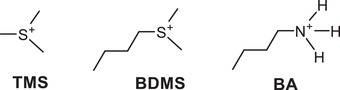
Chemical structures of TMS, BDMS, and BA.

Prior to the introduction of the sulfonium cation into halide perovskites, there remained a need to investigate the polarity of the sulfonium moiety, in conjunction with solution processing to produce PSCs. Previously reported TMS^3^ was selected as a candidate of sulfonium (S^+^), with the expectation of the formation of (TMS)_3_PbI_3_ as a light harvester. Unfortunately, TMS exhibited very poor solubility in typical solvents such as a mixture of dimethylformamide (DMF):dimethylsulfoxide (DMSO) or DMF alone. Although TMS was dissolved by increasing the ratio of DMSO to DMF, the resultant solvent ratio deviated from the typical condition for fabricating efficient PSCs. The relatively higher dielectric constant (ε = 46.7) for DMSO (vs 36.7 for DMF)^4^ required to dissolve TMS suggests that the presence of the polar solvent would play an important role in the solution processing of aprotic polar cations like TMS. The resulting color of the perovskite film obtained by two‐step processing was yellow, corresponding to a reported bandgap of 3.1 eV,^3^ leading to an extremely low PCE of below 1% (Figure S1, Supporting Information).

Based on our results regarding the solubility of the sulfonium moiety, butyldimethylsulfonium iodide (BDMSI) was designed and successfully synthesized using a nucleophilic (S_N_2‐type) substitution reaction (Figure S2, Supporting Information).^5^ Because the amount of guest cations introduced in halide perovskites is crucial to determining device performance, the molar ratio of BDMS was varied to 1, 2, 3, and 4 mol% with respect to MA. It should be added that the device performance was not optimized for this step, as it is more important to find the approximate ratio ensuring both PCE and stability. In general, it was found that the greater the amount of guest cations, the larger the loss in device efficiency (Figure S3, Supporting Information). An increase in the amount of large cations may induce the partial formation of 2D perovskites, which offsets the advantages of 3D perovskites in terms of charge transport and solar capture, resulting in decreased efficiency.^6^ Considering the negative effects of the greater amount of cations on PCE, further study focused on the composition ratio of (BDMS)_0.02_(MA)_0.98_PbI_3_ (denoted as target).

To investigate the surface properties of the resultant perovskite films, X‐ray photoelectron (XPS) spectra and scanning electron microscopy (SEM) were employed. As shown in **Figure** **2** and Figure S4 in the Supporting Information, the core‐level spectra of S 2p, Pb 4f, and I 3d are recorded by XPS. In the case of target perovskite film, the presence of sulfur atoms was reflected in the S 2p spectrum. Judging from the different binding energies of S 2p peak, depending on the type of oxidation state or chemical environment, the binding energy shown at 165.3 eV can be assigned to the sulfonium cation (R_3_S^+^) (cf. S atom (≈163 eV) and O=S=O with electron‐withdrawing atoms (≈168 eV)).^7^ From the comparison of the surface morphology of pristine to target perovskite films, target film showed the smaller crystalline grains than pristine film with pin‐holes free (Figure 2). The cross‐views of SEM images indicate the similar thickness (300–350 nm) for pristine and additive‐treated light‐harvesting perovskite layers (Figure S5, Supporting Information). It is interesting to note that even a small change in the molar ratio of target cations to MA (0.02:0.98) resulted in the change in surface morphology of pristine MAPbI_3_ perovskite film. In addition, the different surface morphology was obtained depending on the molecular structures of cations (cf. Figure 2d vs Figure S5 in the Supporting Information). Considering the same butyl chain length employed for both BDMS and BA, the generic electronic characteristic of atoms introduced on the positive charge might induce the different interactions between precursors in perovskite solution, resulting in the different morphology.

**Figure 2:**
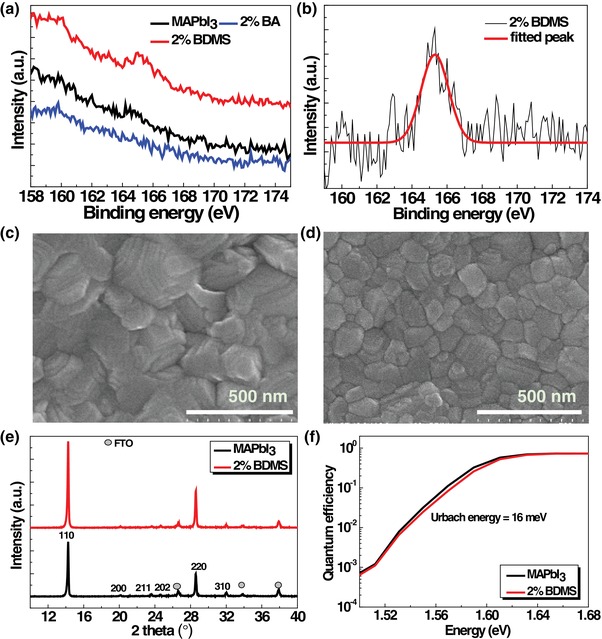
a) XPS core‐level spectra of S 2p for perovskite films and b) fitted XPS spectrum. Top view of SEM images of c) 3D MAPbI_3_ and d) (BDMS)_0.02_(MA)_0.98_PbI_3_ perovskite films. e) XRD patterns of perovskite films. f) Subgap QE spectra of PSCs sensitized with 3D MAPbI_3_ and (BDMS)_0.02_(MA)_0.98_PbI_3_.

Regarding the smaller grains observed from BDMS‐treated perovskite film (Figure 2d), BDMS might be considered as an agent which controls the crystal growth of MAPbI_3_ from the colloid state.^8^ From the result of solubility test showing higher polarity of sulfonium (S^+^) derivatives (vs MAI), BDMS seems to attract the polar solvent of DMSO in perovskite precursor solution. The interaction between BDMS and DMSO might give rise to somewhat smaller grains because DMSO is known to facilitate the growth of perovskites by making a complex of PbI_2_···DMSO···MAI.^9^ Nonetheless, another possible explanation is that the small crystallites are ascribed to the larger cations, which have been reported to form 2D perovskites.^10^ Related research^6^ have shown that the small 2D crystallites formed on top of 3D MAPbI_3_ layer. As potential evidence for the formation of thin 2D perovskites, diffraction peaks appearing in the low‐angle region were suggested. In this regard, further structural analysis was performed by X‐ray diffraction (XRD) analysis. As shown in Figure 2e, pristine 3D MAPbI_3_ followed the reported crystalline structure of the tetragonal phase. The major diffraction peaks appeared near 14°, 24°, 28°, and 32° (2θ), which corresponds to the (110), (202), (220), and (310) lattice planes, respectively. The calculated lattice parameters (Table S1, Supporting Information) indicated the values of *a* = 8.777 Å and *c* = 12.567 Å. Based on the comparison of the characteristic peak position at (110) lattice plane, XRD pattern of the modified perovskites with BDMS remained consistent with that of pristine 3D MAPbI_3_ without lattice expansion or contraction of MAPbI_3_ matrix. The evidence of forming 2D perovskite was not found from this observation through XRD. The results from previous work have claimed that the extremely thin 2D perovskites comprising 2D/3D mixed phase or hybrids formed with the low concentration of larger cations could not be resolved with regular XRD.^11^, ^12^ The subgap quantum efficiency (QE) spectra further support the structural similarity between pristine and target perovskites (Figure 2f). A negligible band tail recombination was observed, giving a narrow distribution of Urbach tails (Urbach energy of ≈16 meV) for both pristine and target perovskites.^13^ Regarding the role of such small organic molecules in the formation of perovskites, there might be two assumptions as follows: 1) small molecules could give rise to the compact perovskite film by forming a complex with PbI_2_ and MAI, and consequently, retarding the crystal growth,^14^ and 2) 2D/3D stacking (or mixed) hybrid perovskites could be formed, which imparts the passivation of the surface and/or grain boundaries of perovskites.^11^ Previous experimental results indicate that the former type of complex formation depends on the electron‐donating power of heteroatom ligands.^15^ However, relatively electron deficient sulfonium (S^+^) might not donate a sufficient amount of electrons toward Pb(II) in PbI_2_. Furthermore, the surface morphology of target film showing smaller grains is contrary to the former scenario. Although we cannot expect the exact configuration of formed perovskites, it is evident that BDMS plays an important role in controlling the growth of perovskites. The results of XPS combined with XRD and subgap QE spectra highlight the effect of BDMS on the morphology, which showed the potential evidence for the presence of BDMS on the surface and/or grain boundaries.

Optical phenomena, one of the important features for the high‐efficiency PSCs were investigated using a UV–vis spectrometer. As shown in Tauc plot (Figure S6, Supporting Information), both pristine and BDMS‐driven perovskite films gave an optical bandgap of 1.59 eV (780 nm). As a result, the use of a small amount of larger cations (vs MA) in MAPbI_3_ would not offset the advantage of larger solar capture arising from 3D perovskites (vs excitonic nature in 2D perovskites). The photovoltaic performance was measured under simulated AM 1.5G irradiation. As expected from the material aspects discussed, the addition of BDMS showed little change in the overall efficiency (≈19%) of 3D PSCs, but a slight increase in *V*_oc_ (**Figure** **3**). Detailed discussion is added in Figures S7 and S8 in the Supporting Information.

**Figure 3:**
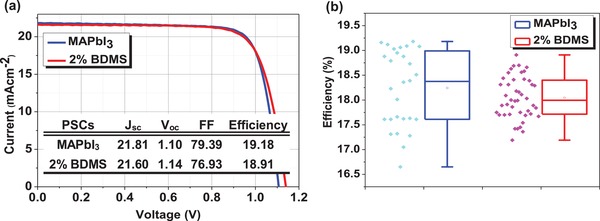
a) *J*–*V* curves of the best PSCs and b) distribution in PCEs of PSCs sensitized with 3D MAPbI_3_ and (BDMS)_0.02_(MA)_0.98_PbI_3_. Device contact was composed of FTO/b‐TiO_2_/m‐TiO_2_/photoactive perovskite/spiro‐OMeTAD/Au.

The stability of target perovskite films on humidity was investigated. The changes in absorbance, emission, and crystal structure of perovskite films were monitored at 80–85% relative humidity (RH) condition. As shown in **Figure** **4**a and Figure S9 in the Supporting Information, target film showed the enhanced tolerance on humidity compared to pristine film. In addition, this higher wet‐fastness of target film seemed to be reflected in the corresponding PSCs (Figure S10, Supporting Information). Nonetheless, it was reported that the PCE values as a simple tracking tool of degradation in PSCs could be affected by many factors including metal contact, delamination, and voids in perovskite layers.^16^ Thus, the properties of perovskite films alone were focused for further analysis. Based on the results of UV–vis spectra, pristine film almost fully degraded within ≈100 h, while target film retained ≈80% of the initial solar capture. Decomposition of 3D perovskite through the formation of PbI_2_ was supported by XRD analysis (Figure 4b; Figure S11, Supporting Information). Related research showed that the degradation under humid condition could take place through the diverse pathways depending on the environmental parameters, thus resulting in different degradation products such as PbI_2_, hydrated perovskites, and/or gaseous products.^17^, ^18^ In conjunction with the formation of the volatile products, it was pointed out that the key active species that degrade perovskites might not be water but rather hydroxide (OH^−^) and hydroxyl radical generated from processing.^19^ Regardless of the types of active species to decompose perovskites, it would be expected that the acid–base reaction^20^ occurs by donating H^+^ from the protic organic cations (e.g., RH_3_N^+^) to the active base species, thus releasing the volatile compounds like methylamine (CH_3_NH_2_).

**Figure 4:**
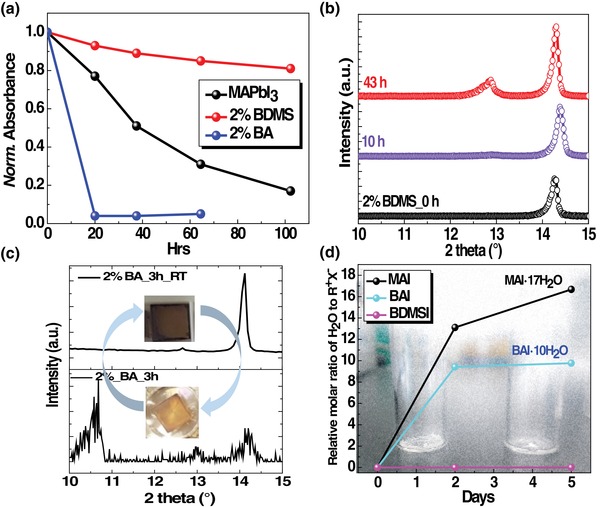
a) Time evolution of absorbance of pristine and modified perovskite films. X‐ray diffraction patterns of BDMS‐based b) and BA‐treated c) perovskite films. d) Water‐uptake of precursors of cations obtained from quantitative NMR analysis. Here, all samples were stored at 80–85% RH and room temperature under dark condition without encapsulation. The notation of BA_3h_RT in (c) refers to the storage of samples for 3 h at 85% RH followed by storage at ≈20% RH.

Regarding the origin of enhanced humidity stability for the additive‐treated 3D MAPbI_3_ perovskites, it was reported that the morphology, crystallization, and/or hydrophobicity of perovskites could affect the interaction between H_2_O and the perovskite layer.^21^, ^22^ For example, ammonium cations having long alkyl chains or aromatic groups was introduced to impart the hydrophobicity on perovskite film.^23^ According to previous studies, the improved humidity stability of the BDMS‐treated perovskite film might be simply ascribed to the hydrophobic butyl chain (vs methyl chain of MA ion). However, we also pointed out the chemical environment near the nitrogen atom of ammonium cations. Taking into consideration the molecular structure of ammonium cation of MA, our expectation was that the fully deprotonated cations employed into perovskites might significantly reduce the chance of the interaction of perovskites with water molecules. Specifically, it is likely that the aprotic cations do not permit the acid–base reaction, thus increasing humidity stability of perovskites.

In order to further investigate the effect of protons attached to cations on the hydration of perovskites, BA was chosen as a corresponding candidate of BDMS. Notably, BA‐treated perovskite film showed the significantly low wet‐fastness with respect to the corresponding target film. Specifically, after 5 h of storage under 85% RH, the shape and emission wavelength of BA‐based film were completely changed from the initial feature (Figure S12, Supporting Information). Based on the XRD results and digital images taken (Figure 4c), the measured emission feature might be related to the hydrated form (*n*H_2_O·MAPbI_3_) of perovskite.^17^ Assuming the virtually similar molecular geometry except the localized cation moiety between BA and BDMS, this finding led us to focus on the properties of cations introduced in perovskites. The water‐uptake of each cation stored under 85% RH condition was tracked through NMR analysis. As shown in Figure 4d and Figure S13 in the Supporting Information, BA with three protons near the nitrogen atom easily absorbed H_2_O molecules. MAI with three protons but a shorter methyl chain showed the greater water‐uptake than BA. On the other hand, the nonprotonated derivative of BDMS showed the unchanged spectrum within current tracking time range. The more acidic nature of BA (vs BDMS) was evident, judging from the incredibly increased water‐uptake of BA. This finding further supports that the protic BA would be vulnerable to the acid–base reaction. Consequently, the generic chemical nature of guest cations seemed to affect humidity stability of perovskite films assembled with those cations. Nonetheless, it is interesting to note that the stability of BA‐treated perovskites on humidity was even lower than that of pristine perovskites (Figure 4a). As shown in Figure 4c, the BA‐based perovskite film seems to undergo a reversible reaction between the black and hydrated phases of perovskites, especially at the early stage of degradation. The black phase of perovskite was realized after removal of samples from the high humidity condition. However, after some critical time of storage under high humidity, the black phase of perovskite was not retrievable (Figure S14, Supporting Information). Considering the more hydrophobic (less acidic) character of BA (vs MA), the surface treatment of 3D perovskite film with BAI was performed with the two different molarities. Such hydrophobic character of BA was supported by the fact that the increased amount of BA clearly slowed down the degradation of perovskite film in humid air (Figure S15, Supporting Information). The reason for the unexpected low wet‐fastness for the BA‐treated perovskite film (vs pristine film) is still unclear, considering the more hydrophobic (less acidic) nature of BA (vs MA). Nonetheless, based on the assumption that BA exists on the increased numbers of grain boundaries (vs pristine films), insufficient coverage of the perovskite surface might induce the abrupt collapse of perovskite phase, which is caused by the acid–base reaction between the basic degradation‐initiator and protic BA. Therefore, it can be concluded that the configuration of perovskites combined with acidic nature of BA may not produce the desirable hydrophobic surface enough to protect 3D perovskite against humid environment, particularly in the case of using the small amount of guest cation.

As an approach to theoretically study the functionalized perovskite surfaces with small molecules, density functional theory (DFT) calculations inspired by the work of Yang et al.^24^ were performed. Although many surfaces in perovskites could be expected depending on the type of termination, defect, and/or orientation,^25^ current study adopted the (100) MAPbI_3_ surface which has been suggested to be stable than other surfaces. **Figure** **5**a shows the optimized structures of the functionalized surfaces of MAPbI_3_ with additives. The stable conformations of additives adsorbed on the (100) MAPbI_3_ surface had the aliphatic hydrophobic chains pointing upward, while the polar group of guest cation prefers to face perovskite surfaces. Furthermore, due to the limited free volume at the functionalized MAPbI_3_ surfaces which can allow the guest cations to rotate, this arrangement remained consistent after adsorbing water to produce the hydrated perovskite surfaces (Figure 5b). Following adsorption of water, the oxygen atom of water made a bond with the dangling Pb atoms on the (100) surface of MAPbI_3_. On the other hand, it is likely that the 3D geometry near heteroatoms of the guest cations significantly change the adsorption energetics of water on the modified perovskite surfaces, giving an adsorption energy of −0.58 eV and −0.34 eV for the BA‐ and BDMS‐treated perovskite surface, respectively. Interestingly, the adsorption energy of water on the BA‐driven perovskite was virtually comparable to pristine MAPbI_3_ surface (−0.55 eV). Considering that the polar part of additives was located in troughs formed by neighboring lead‐halide octahdera, it is expected that essentially the same geometry arising from the protic ammonium cores dominates the conformations (tilting) of the BA‐treated surface. Unlike the protic ammonium‐treated perovskites, the much smaller adsorption energy of water for the BDMS‐treated perovskite surface might benefit from the bulky size of sulfur atom, making the functionalized MAPbI_3_ surface more strained compared to pristine or BA‐treated perovskite surface (Figure 5a). The resultant molecular configuration could make water difficult to land on Pb atoms on the BDMS‐treated perovskite surface, resulting in higher wet‐fastness (Figure 5b). This finding suggests a steric effect of guest cations as one of the factors affecting moisture stability of the surface‐modified perovskites, especially regarding the first step for the hydration of perovskites.

**Figure 5:**
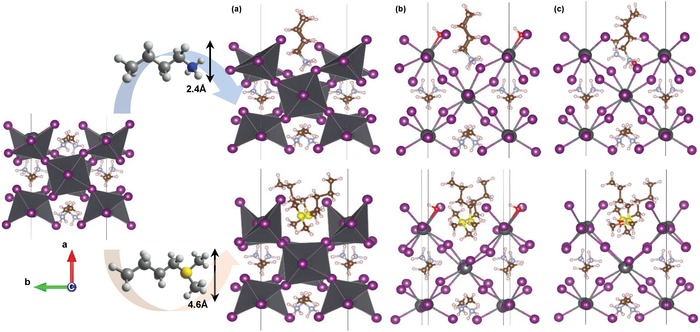
a) The optimized (100) surfaces of MAPbI_3_ perovskites treated with guest cations followed by b) the adsorption of water. c) The relaxed (100) surfaces of modified perovskites when water is closer to the heteroatoms of the guest cations. Here, upper and lower images correspond to the modified perovskites with BA and BDMS, respectively. Far left side of image is the (100) surface of pristine MAPbI_3_. (For the atoms in (a)–(c), Pb (gray), I (violet), S (yellow), N (blue), C (brown), and O (oxygen).

To investigate an effect of protons on the energetics when water molecule is infiltrated toward bulk MAPbI_3_ matrix, water was manually located closer to heteroatoms of additives. The initial energy value of the hydrated perovskite surface (Figure 5b) was referenced for the comparison of the water‐infiltrated perovskite system (Figure 5c). The infiltration process requires the activation energy to break the Pb—O bonds formed at the initial hydrated perovskite surface. Compared to BDMS‐driven perovskite, the activation energy of BA‐treated perovskite was reduced by ≈0.1 eV, which might be associated with the stabilization of system by the hydrogen bonding between the proton of BA and oxygen atom of water (Figure 5c; Figure S16, Supporting Information). In the case of BA‐driven perovskites, it should be noted that the other conformations are possible due to the three possible H‐bond sites from BA (Figure S16, Supporting Information). However, acid–base reaction was not observed in the presence of water, judging from the absence of oxonium ion (H_3_O^+^). Previous research on the ab initio statics and dynamics showed that hydroxide or hydroxyl radical is the main culprit of degradation and induced the instant hydrogen abstract from the protic cations (e.g., MA), consequently producing the degradation product of gaseous methylamine.^25^ Similarly, it could be expected that the proton of BA is instantly transferred to the unstable radical and/or hydroxide from the molecular arrangement shown in Figure 5c. From these results, it is evident that the BA‐modified perovskite is vulnerable to moisture from the view of geometry of ammonium cores as well as its protic chemical nature.

In summary, BDMS cation was successfully synthesized and assembled in the form of (BDMS)_0.02_(MA)_0.98_PbI_3_ into PSC. The effect of the addition of BDMS on the photovoltaic performance was very small, but humidity stability was significantly increased. From the similar butyl chain geometry of BDMS to BA, the basicity of cations along with hydrophobic alkyl (or aromatic) chains should be considered when engineering the large cations for use in perovskites. The benefit of hydrophobic butyl chains was weakened when a small amount of guest cations was used, resulting in the unexpected low wet‐fastness for the BA‐derived perovskite film. Such phenomenon should be augmented by the modeling study, which is designed to examine the configuration at the different perovskite surfaces by varying the amount of protic ammonium cations. DFT calculations on BDMS‐functionalized (110) MAPbI_3_ surface further emphasized the molecular aspect of bulky aprotic feature on moisture stability, by giving the lower adsorption energy of water and higher activation energy for the infiltration of water. Owing to the prominent photovoltaic performance and stability of BDMS‐driven PSCs, sulfonium cations are considered as the suitable cations for the next alternatives to current ammonium cations.

## Conflict of Interest

The authors declare no conflict of interest.

## Supporting information

Supporting Information
