# Supplementary material for: Enhanced Moisture Stability by Butyldimethylsulfonium Cation in Perovskite Solar Cells
Source: Adv Sci (Weinh). 2019 Dec 13;7(3):1901840. doi: 10.1002/advs.201901840 (PMC7001633; doi:10.1002/advs.201901840)
Supplement: Supplementary file 1 — Supporting Information [file ADVS-7-1901840-s001.pdf]

Copyright WILEY-VCH Verlag GmbH & Co. KGaA, 69469 Weinheim, Germany, 2019.

ADVANCED SCIENCE
Open Access

Supporting Information

for *Adv. Sci.,* DOI: 10.1002/advs.201901840

Enhanced Moisture Stability by Butyldimethylsulfonium Cation in Perovskite Solar Cells

*Bohyung Kim, Maengsuk Kim, Jun Hee Lee, and Sang Il Seok**

Supporting Information

Enhanced moisture stability by butyldimethylsulfonium cation in perovskite solar cells

*Bohyung Kim, Maeng-Suk Kim, Jun Hee Lee, Sang Il Seok**

Experimental Procedure

Materials: All reagents were purchased from Sigma-Aldrich, TCI, or Greatcellsolar. All chemicals were of reagents-grade quality and solvent purchased from commercial suppliers were used without further purificiation.

Synthesis of cations

MAI: Into an ice-cooled flask were placed methylamine. Hydriodic acid was then added dropwise and reaction mixture was stirred for 2.5 h while the solution was kept at 0° C. The solvent was then evaporated. The resulting solid was recrystallized from the ethanol to produce a white color. Yield: 20%, ^{1}H NMR (400 MHz, DMSO-*d6*) δ = 7.50 (s, 1H), 2.38 (s, 1H). 9.28. ^{13}C NMR (400 MHz, DMSO-*d6*) δ = 25.01.

BDMSI: 1-iodobutane and dimethyl sulfide was stirred overnight. The solid obtained was recrystallized from the acetone to produce a white solid. Yield: 30%, ^{1}H NMR (400 MHz, $CDCl_3$) δ = 9.28 (t, 2H), 3.40 (s, 6H), 1.83 (m, 2H), 1.58 (m, 2H), 1.02 (t, 3H). ^{13}C NMR (400 MHz, $CDCl_3$) δ = 42.68, 26.11, 25.69, 21.60, 13.63.

Preparation of perovskites films: A 1.13 M reference solution with PbI_2 and MAI was prepared in a mixed solvent of dimethylformamide and dimethyl sulfoxide with a volume ratio of 2:8 followed by stirring at 65 ℃ for 1 h. The resulting solution was spin coated on the substrate by gradually increasing rpm through three continuous steps (first step at 500 rpm for 5 s, second step at 1000 rpm for 5 s, third step at 5000 rpm for 50 s). A 1000 μL of anti-solvent of chlorobenzene was poured at final third step followed by annealing at 100 ℃ for 10 min to afford a reference of 3D $MAPbI_3$. Introduction of BDMS into the $MAPbI_3$ matrix was done by variations in molar ratio of MAI to the BDMS in precursor solution. Here, A-site cation of MA

in ABX_3 perovskites was partially substituted with BDMS, resulting in a chemical formula of $(BDMS)_x(MA)_{1-x}PbI_3$.

*Fabrication of PSCs***:** An FTO glass plate (F-doped SnO_2, TEC-8, 8Ω, Pilkington) was etched with zinc powder and HCl to prepare cathode. The patterned FTO was cleaned in a detergent solution using an ultrasonic bath for 20 min, followed by washing with acetone and ethanol. A compact blocking layer of TiO_2 (b-TiO_2) was first deposited onto the FTO glass by spray pyrolysis, with a diluted solution of Ti(iv) bis(ethylacetoacetato)-diisopropoxide (75 wt.% in isopropanol). Here, the FTO glass kept at 500 °C for sintering of this layer. A diluted solution of TiO_2 paste (aver. diameter of 50 nm, anatase) was prepared using a mixture solution of terpineol and 2-methoxy ethanol (wt.% of 1:3.5). A mesoporous TiO_2 layer (m-TiO_2) was then coated on the preformed b-TiO_2 layer by spin-coating of 85 μL of the prepared solution, followed by sintering at 500 °C for 1 h. After cooling to room temperature, UV-ozone was treated for 20 min. The light harvesting layer of perovskites was fabricated by one-step spin-coating method as mentioned in a section of the preparation of perovskites films. The resulting film was composed of a ~ 100 nm thick m-TiO_2 layer and 300 ~ 350 nm thick light harvesting perovskite layer. A hole transporting layer (HTL) was spin-coated onto the perovskite layer at 3,000 rpm for 30 s using a spiro-OMeTAD in chlorobenzene solution (100 mg in 1.1 mL) containing additives, which are composed of 39 μL of TBP, 23 μL of LiTFSI (from stock solution of 0.52g mL^{-1} in acetonitrile), 10 μL of Co(II)TFSI (from stock solution of 0.38 g mL^{-1} in acetonitrile. A cathode contact was finally formed on top of the HTL by a thermal evaporation of gold under a negative pressure down to 1 x 10^{-7} Torr. Thickness of the resultant layer of gold was a 70 nm.

Analytical instrumentation and measurements

Structure confirmation: ^{1}H NMR and ^{13}C NMR spectra were recorded on a 400 MHz (Bruker Advance III HD) or 600 MHz (Agilent VNMRS) spectrometer using DMSO-d_6 or

$CDCl_3$ as a solvent. For quantitative analysis, solvents of DMSO-d_6 and acetonitrile were used as a blank and internal reference, respectively.

Characterization of perovskite films: The XRD patterns were obtained using a Rigaku D/MAX2500/PC diffractometer with a Cu Kα (λ = 1.5406 Å) as an X-ray source operated at 40 kV and 200 mA. Top-, cross images, and thickness of thin layers were acquired using a Hitach S-4800 equipped with a cold field emission gun (FEG) as an electron source. UV-Vis spectra were recorded on a JASCO V-780 UV-Vis spectrometer. Photoluminescence were obtained using a fluorometer (Fluotime 300, PicoQuant, Germany) equipped with a 520 nm laser. Emission lifetimes were measured by means of time-resolved single photon counting (TCSPC) mode with a 520 nm green laser as an excitation wavelength. X-ray photoelectron spectroscopy (XPS) was recorded on a thermos Fisher Scientific ESCALAB 250XI with a monochromatic Al Kα X-ray source with an excitation energy of 1486.6 eV. The measurement was done without additional etching to prevent reconfiguration of ions in perovskites. Chamber pressure was kept at 1x10-10 Torr during measurement.

Electrochemical measurements: Electrochemical impedance spectroscopy (EIS) were recorded with a frequency response analyzer (FRA) connected to a potentiostat (PGSTAT-30, Autolab, Netherlands) in a frequency range of 0.1 Hz – 10^5 Hz under dark and illumination condition. The bias of 0.5 V and open-circuit voltage were applied for dark and illumination condition. The AC voltage perturbation set to 10 mV.

Photovoltaic performance of PSCs: Photocurrent-voltage characteristic of PSCs were measured using Keithley 2400 source meter under illumination of AM 1.5 G (100 mWcm-2 solar light coming from solar simulator (Oriel Class A, 91195A, Newport, USA) equipped with a 450 W xenon lamp (6280NS, Oriel). The incident light intensity was calibrated using a reference Si solar cell (Newport Oriel, 91150V) to set 1 Sun (1 mW·cm-2). The current-voltage curve of the cell was obtained by applying external bias and the measuring generated photocurrent. The measurement was fully controlled under Solar I-V software. A mask (0.096

cm^2) was covered on the testing cell during photocurrent and voltage measurement. IPCE (incident monochromatic photon to current conversion efficiency) experiments were carried out using a system (IQE 200B, Oriel, USA) equipped with a 100 W xenon lamp (6252NS, Oriel) as a light source connected to a monochrometer. Calibration of incident light was performed using a Si photodiode (IQE-SAMPLE-SI, Newport, USA). Monochromatic quantum efficiency was recorded at short circuit conditions under AC mode with white-light bias. chopping speed of AC was set to 25 Hz.

Stability test: Humidity stability of PSCs and photo active layer of perovskites was measured by tracing change in optical and crystal structure. All samples were stored at 85% RH and 25 ℃ under dark condition using a thermos-hygrostat chamber. All PSCs were tested without encapsulation.

DFT calculations: The density functional theory (DFT) simulations were performed within the generalized gradient density approximation (GGA) as implemented in the Vienna ab initio simulation package. The electron-ion interactions were described using the projector-augmented-wave (PAW) method[1] with the valence states of 5d10 6s2 6p2 for Pb, 5s2 5p5 for I, 2s2 2p2 for C, 2s2 2p3 for N, 2s2 2p4 for O, 3s2 3p4 for S, and 1s1 for H. The Perdew-Burke-Ernzerhof (PBE) functional was used for GGA exchange correlation potential.[2] The dispersive van der Waals interactions are included by using the nonlocal van der Waals (vdW) density functional of vdW-optB86b.[3] To simulate MAI-, BAI, BDMSI-terminated (100) tetragonal $MAPbI_3$ surface, a kinetic energy cutoff of 400 eV and 1x4x2 k-points for Brillouin zone integration were used. All the atomic positions and lattice parameters are relaxed until the total energy is converged within 10^{-4} eV

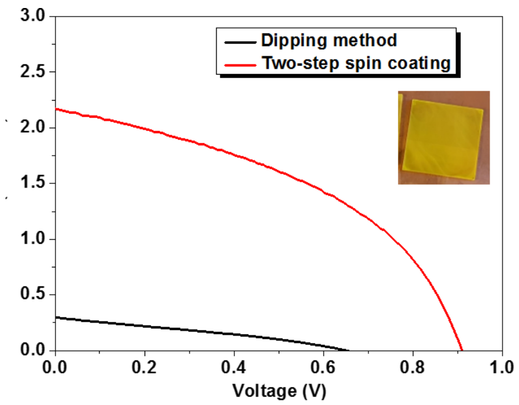

Figure S1. *J-V* curves of PSCs assembled with trimethylsulfonium lead iodide (TMSI) and PbI_2 by two-step solution processing.

Table S1. Lattice parameters, FWHM (full width half maximum), and average crystal sizes obtained from XRD analysis.

Perovskites	2 theta	*cal.* lattice parameter	FWHM	*cal.* crystallites size
$MAPbI_3$	14.26	8.777	0.13058	61.31
2% BDMS	14.26	8.777	0.13542	59.12

The lattice constants for tetragonal phase were determined by following equation:

$$\frac{1}{d^2} = \frac{h^2 + k^2}{a^2} + \frac{l^2}{c^2}$$

where d is interplanar spacing and a, c are lattice constants and h,k,l represent miller indices.

Table S2. Photoluminesence decay parameters of perovskite films.

Perovskites	τ_1 (ns)	f_1 (%)	τ_2 (ns)	f_2 (%)	$\langle\tau\rangle$ [a]
$MAPbI_3$	16.53	23.79	58.93	76.21	48.84
2% BDMS	18.96	20.83	82.91	79.17	69.59

[a] $\langle\tau\rangle = \sum f_i \tau_i$

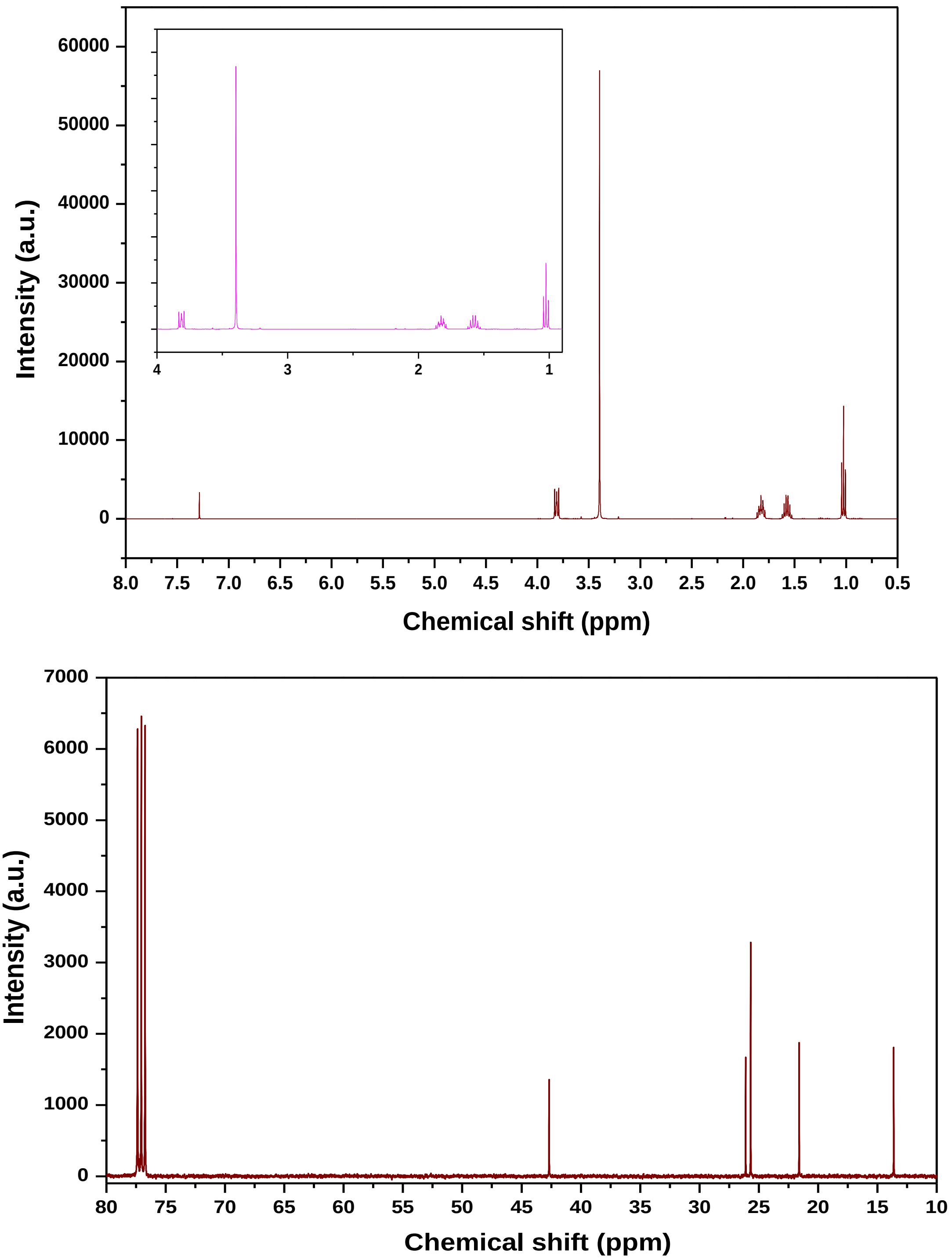

Figure S2. ^{1}H (upper) and ^{13}C (lower) NMR spectrum of BDMSI (butyldimethylsulfonium iodide).

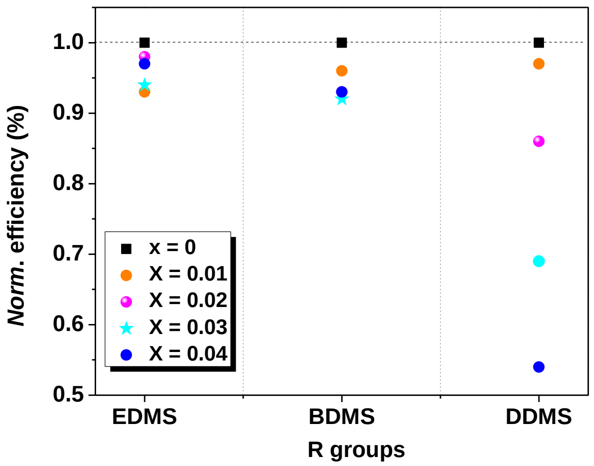

Figure S3. Variations in efficiencies of PSCs based on the $((CH_3)_2RS)_x(MA)_{1-x}PbI_3$ depending on the relative amount of each cation employed. Here, R means ethyl, butyl, and dodecyl for EDMS, BDMS, and DDMS, respectively.

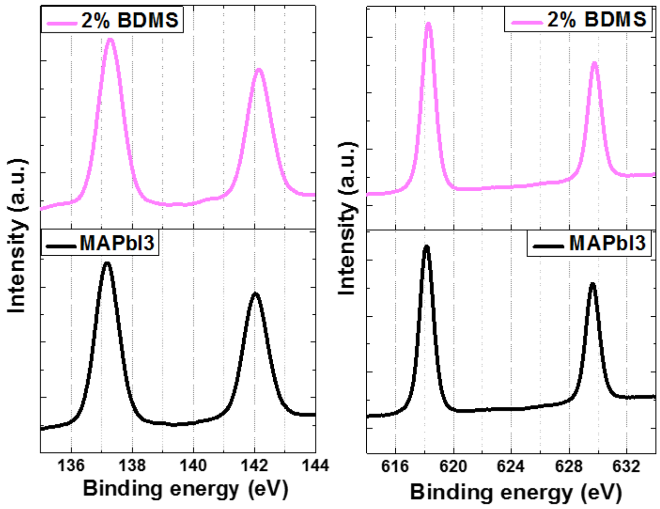

Figure S4. XPS core-level spectra of Pb 4f (left) and I 3d (right) for pristine and $(BDMS)_{0.02}(MA)_{0.98}PbI_3$ perovskite films.

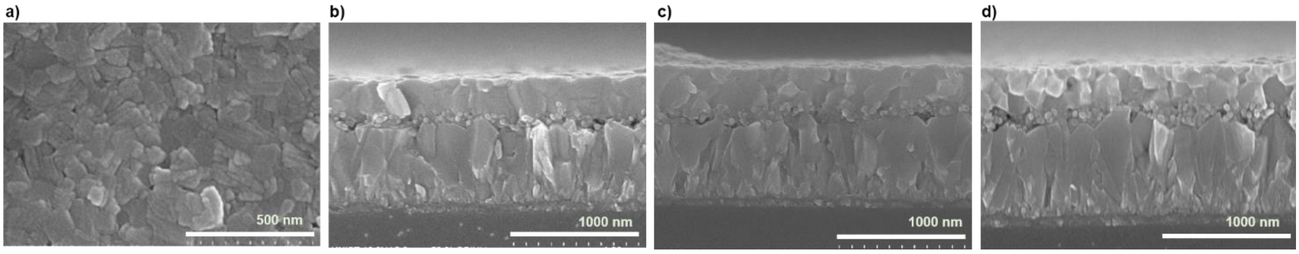

Figure S5. SEM images of perovskite films. The top view of $(BA)_{0.02}(MA)_{0.98}PbI_3$ (a) and cross view of 3D $MAPbI_3$ (b), $(BDMS)_{0.02}(MA)_{0.98}PbI_3$ (c), and $(BA)_{0.02}(MA)_{0.98}PbI_3$ (d) perovskite films.

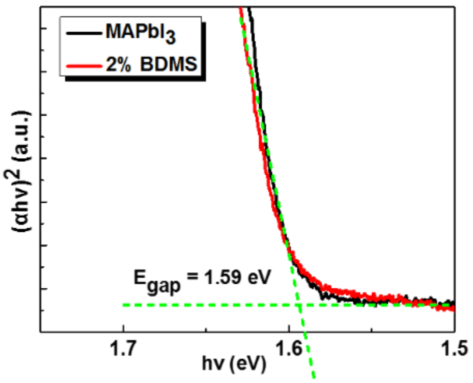

Figure S6. Tauc plot of 3D $MAPbI_3$ and $(BDMS)_{0.02}(MA)_{0.98}PbI_3$ perovskite films.

Considering the effect of charge recombination on the voltage parameter into consideration[4], recombination kinetics were investigated in view of both perovskites alone and interfaces of PSCs. Time resolved photoluminescence spectra (TRPL) were recorded to monitor recombination of photo-generated carriers in perovskite film by using a time-correlated single photon counting (TCSPC) system. The decay curves obtained through TCSPC were fit to a bi-exponential decay function: $F(t) = B1 \cdot exp(-t/\tau_1) + B2 \cdot exp(-t/\tau_2)$, where τ is lifetime constants (Figure S7 and Table S2). Among the two lifetime constants, the fast-decay component was assigned to non-radiative recombination, while the slow-decay components arose from the radiative recombination process. Notably, recombination kinetics of pristine and BDMS-treated perovskite film were dominated by the radiative recombination with a larger faction of τ_2 value. Furthermore, the value of $<\tau>$ increased by ~ 43% (from 49 to 70 ns) by the introduction of BDMS in $MAPbI_3$ perovskite. Assuming that the trap-assisted recombination is mainly responsible for the non-radiative recombination processes, it seems that BDMS played an important role in giving rise to the passivation effect (healing) of surface-defective 3D perovskites, resulting in the reduced fraction of τ_1 value in decay kinetics.[5] As another approach to study kinetics, electrochemical properties at the interfaces of PSCs were measured under dark and illumination condition by EIS. As shown in Figure S8 indicating Nyquist plots, it showed a somewhat different behavior between pristine and modified PSCs. Assuming that Rs (series resistance) referring to the resistance of FTO is the same for both cells,[6] a large difference at mid- and low frequency regime was found. Here, this resistance is responsible for the recombination resistance (R_{rec}) at the TiO_2/perovskite interface.[7] The value of R_{rec} for the BDMS-based PSCs, which was measured under dark condition, was two-fold higher than that of pristine PSCs (228 Ω *vs.* 459 Ω). This relative trend remained consistent for the EIS result obtained from illumination condition at open-circuit voltage. The small arc feature in very low frequency regime (below ~ 100 Hz) may come from a process involving long relaxation time (*e.g.* ionic movement inside perovskites) although the resolution of this feature is still on-going investigation. Consequently, the result showing the increased R_{rec} provided the evidence of decreased charge recombination at the contact of TiO_2/perovskite, leading to higher V_{oc} values for BDMS-based *versus* pristine PSCs. Concerning the origin of V_{oc} gain in modified PSCs with BDMS, related research pointed out a relationship between the recombination rate and morphology of perovskite film in PSCs.[8] Specifically, the reduced gain size of perovskites caused by additives could be penetrated into *m*-TiO_2 pores, thus enhancing the charge transport at the contact of TiO_2/perovskite. Similarly, BDMS-treated perovskites showing the reduced sizes of grains led to the reduced recombination at the contact of TiO_2/perovskite. Although the exact morphology at each contact is still unclear under current scope of research, introduction of BDMS imparted a passivation effect in PSCs, judging from the electrochemical and TCSPC analysis.

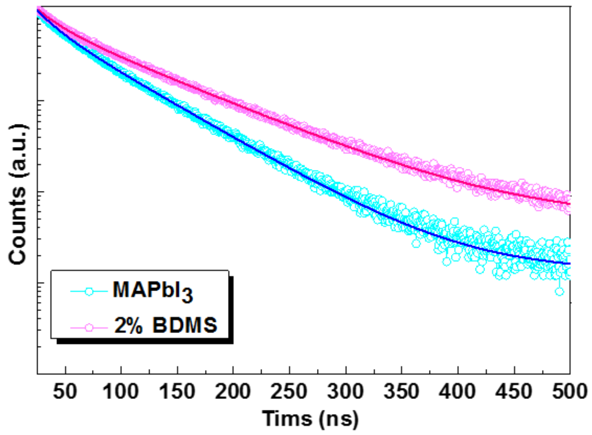

Figure S7. Emission decay curves of 3D $MAPbI_3$ and $(BDMS)_{0.02}(MA)_{0.98}PbI_3$ perovskite films.

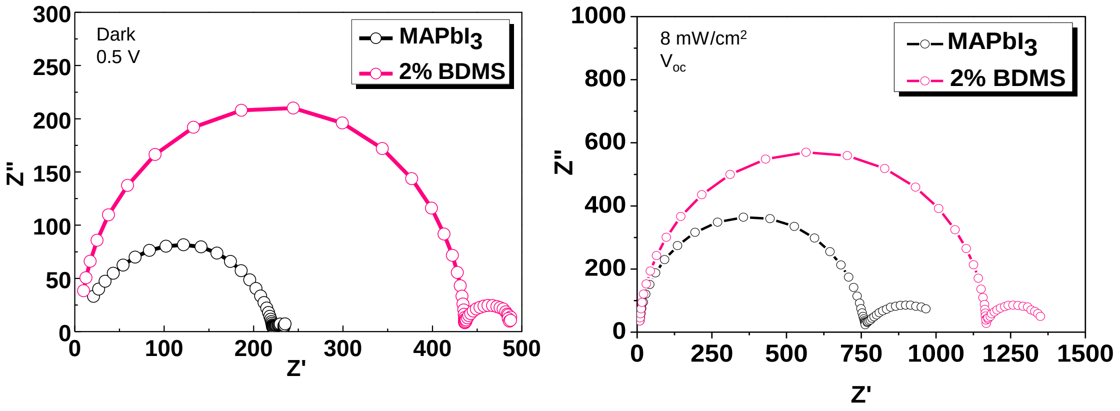

Figure S8. Nyquist phase plot of PSCs sensitized with 3D $MAPbI_3$ and $(BDMS)_{0.02}(MA)_{0.98}PbI_3$. Device contact was composed of FTO/*b*-TiO_2/*m*-TiO_2/photo-active perovskite/spiro-OMeTAD/Au.

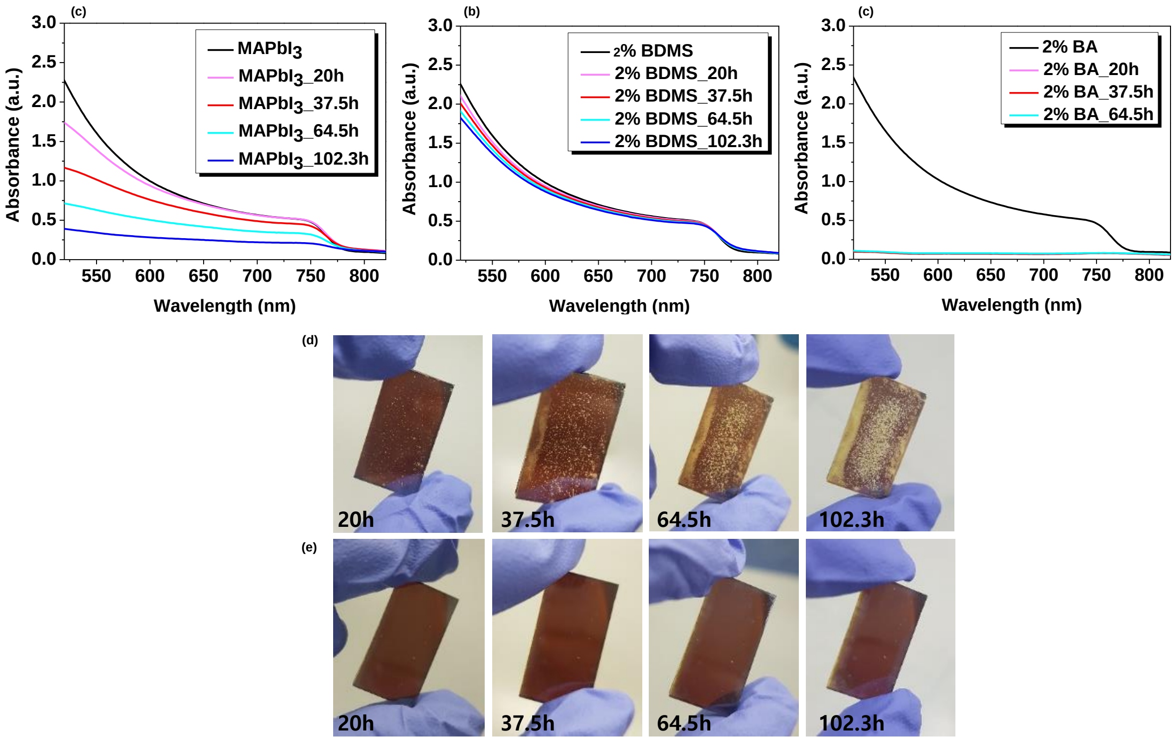

Figure S9. The change in absorbance of perovskite films as a function of time (a-c) and the corresponding digital images for pristine (d) and BDMS-treated (e) perovskite films. All samples were stored under 85% RH and dark condition.

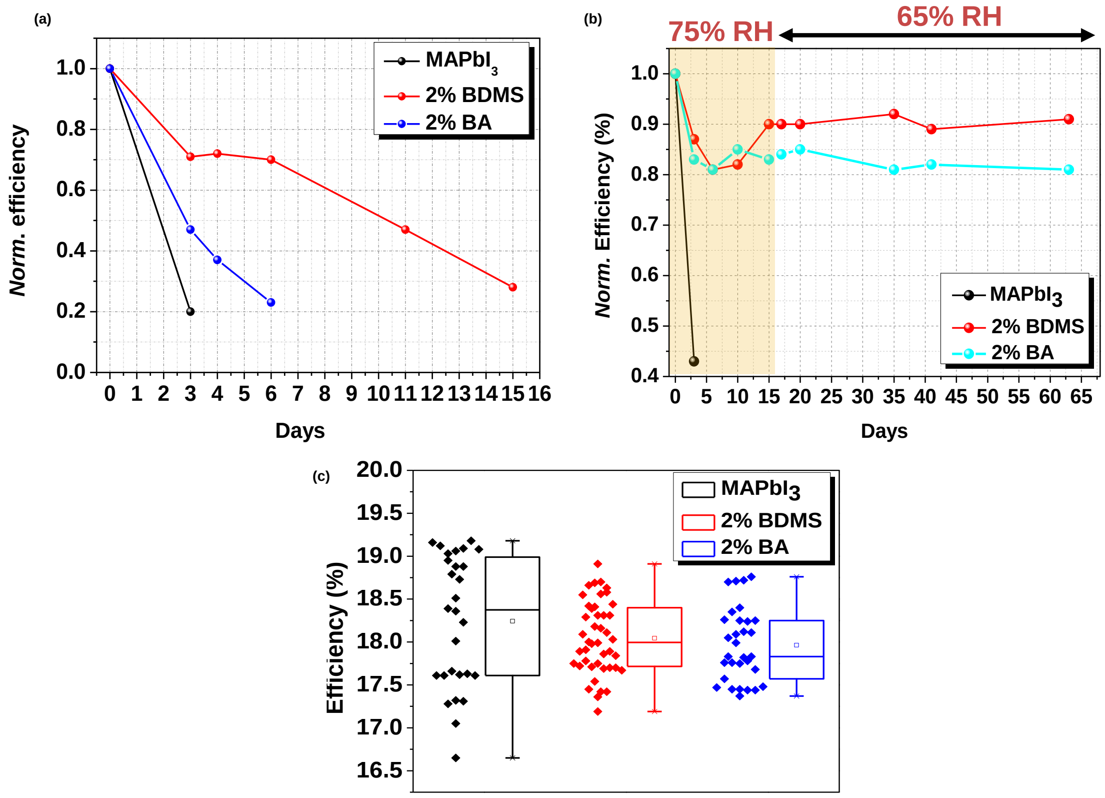

Figure S10. Normalized efficiencies of PSCs sensitized with $MAPbI_3$, $(BDMS)_{0.02}(MA)_{0.98}PbI_3$, and $(BA)_{0.02}(MA)_{0.98}PbI_3$, which were stored under 85% RH (a) and 65% ~ 75% RH (b) condition at room temperature. The initial efficiencies of PSCs before degradation (c).

From Figure S 10b, the slight fluctuation in PCEs was observed at the time in which humid environment was changed from the harsh to milder condition. As mentioned in manuscript, PCE values as a simple tracking tool of degradation in PSCs could be affected by many factors including metal contact, delamination, and voids in the perovskite layers. Assuming that all devices have the similar contact, the value of PCE could be associated with the recrystallization[9-10], filed induced ion movement and/or charge accumulation[11]. In addition, the passivation of defects and/or grain boundaries was claimed from TCSPC result showing the increased lifetimes for the hydrated perovskites.[12]

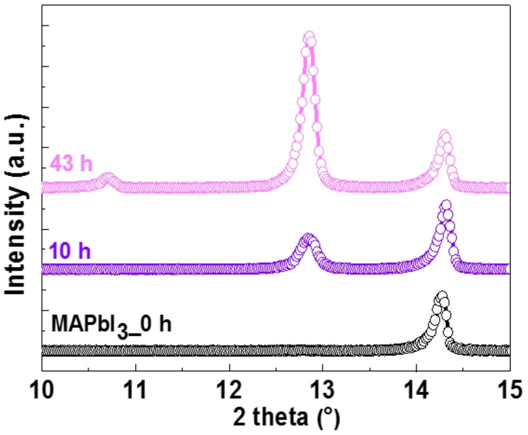

Figure S11. Time evolution of X-ray diffraction patterns of pristine perovskite films.

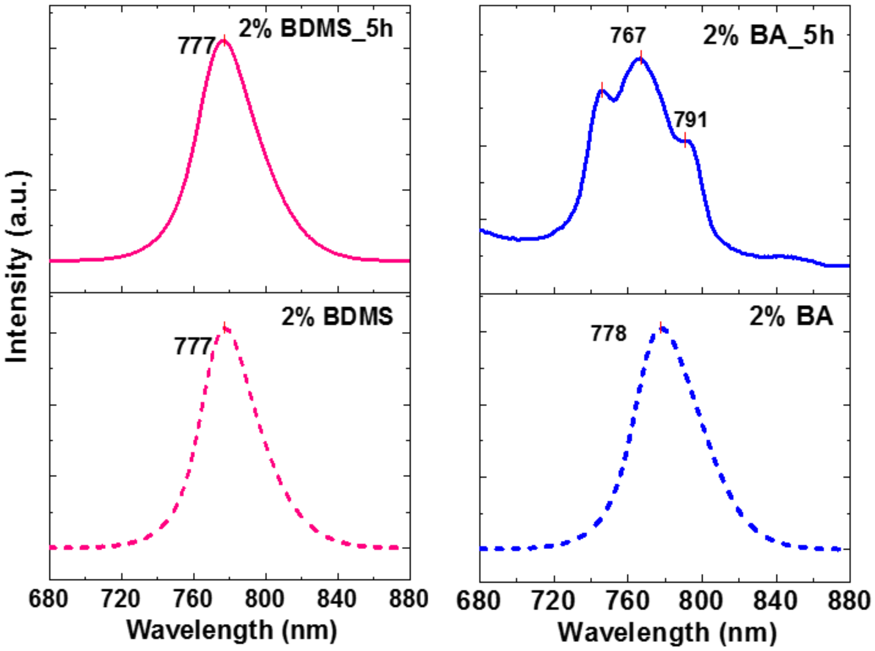

Figure S12. Emission spectra of $(BDMS)_{0.02}(MA)_{0.98}PbI_3$ (left) and $(BA)_{0.02}(MA)_{0.98}PbI_3$ perovskite films (right). Lower and upper graphs were obtained from the fresh film and after 5h storage at 80% ~ 85% RH and room temperature under dark condition without encapsulation, respectively.

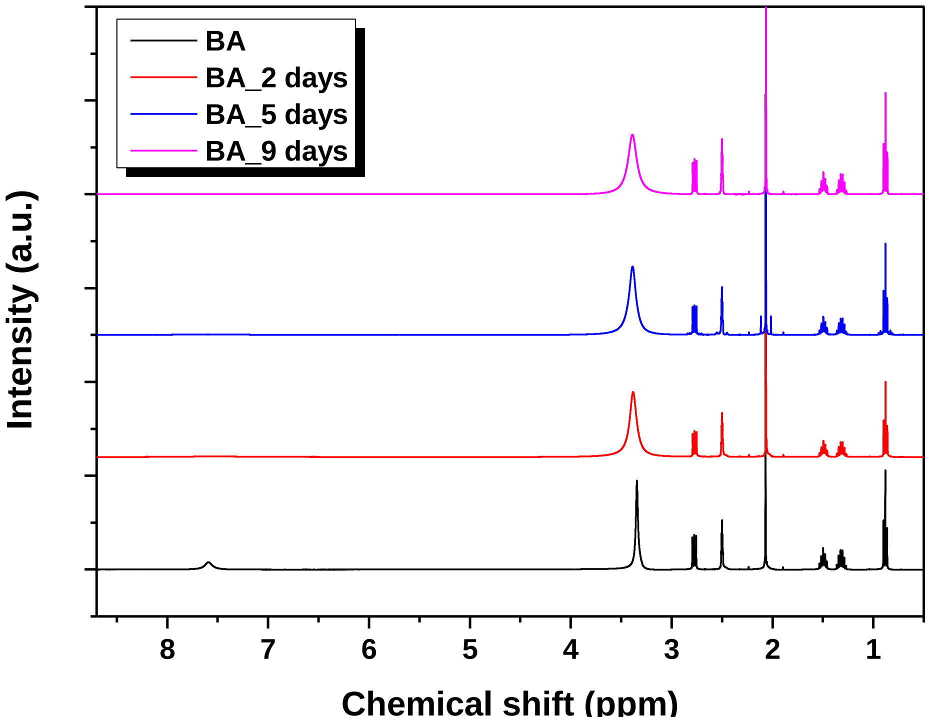

Figure S13. Time evolution of NMR spectra of BA stored under 85% RH at room temperature

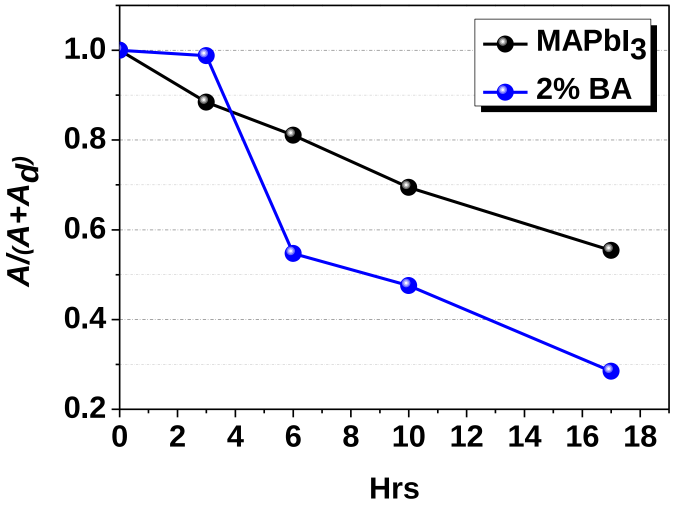

Figure S14. Early time evolution for the crystalline phase of pristine and $(BA)_{0.02}(MA)_{0.98}PbI_3$ perovskite films which were stored under 85% RH at room temperature without encapsulation. Here, A and A_d indicate the integrated area at typical peak ~ 14.26° and peak appearing from PbI_2 and/or hydrated phase due to degradation, which was detected from XRD measurement.

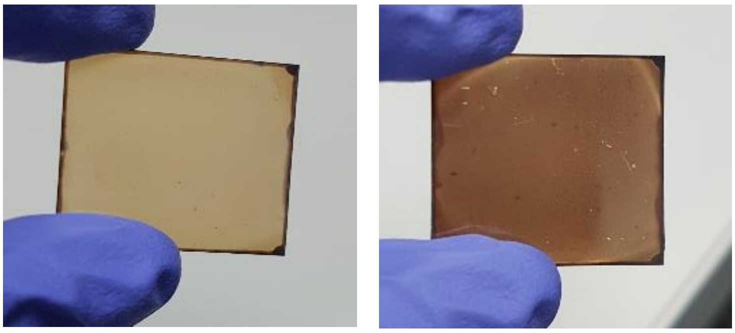

Figures S15. Digital images of surface-treated perovskite films with BA in the case of using an amount of 1mg/1mL (left) and 5mg/1mL (right), which were taken after 3 h of storage under 85% RH at room temperature without encapsulation

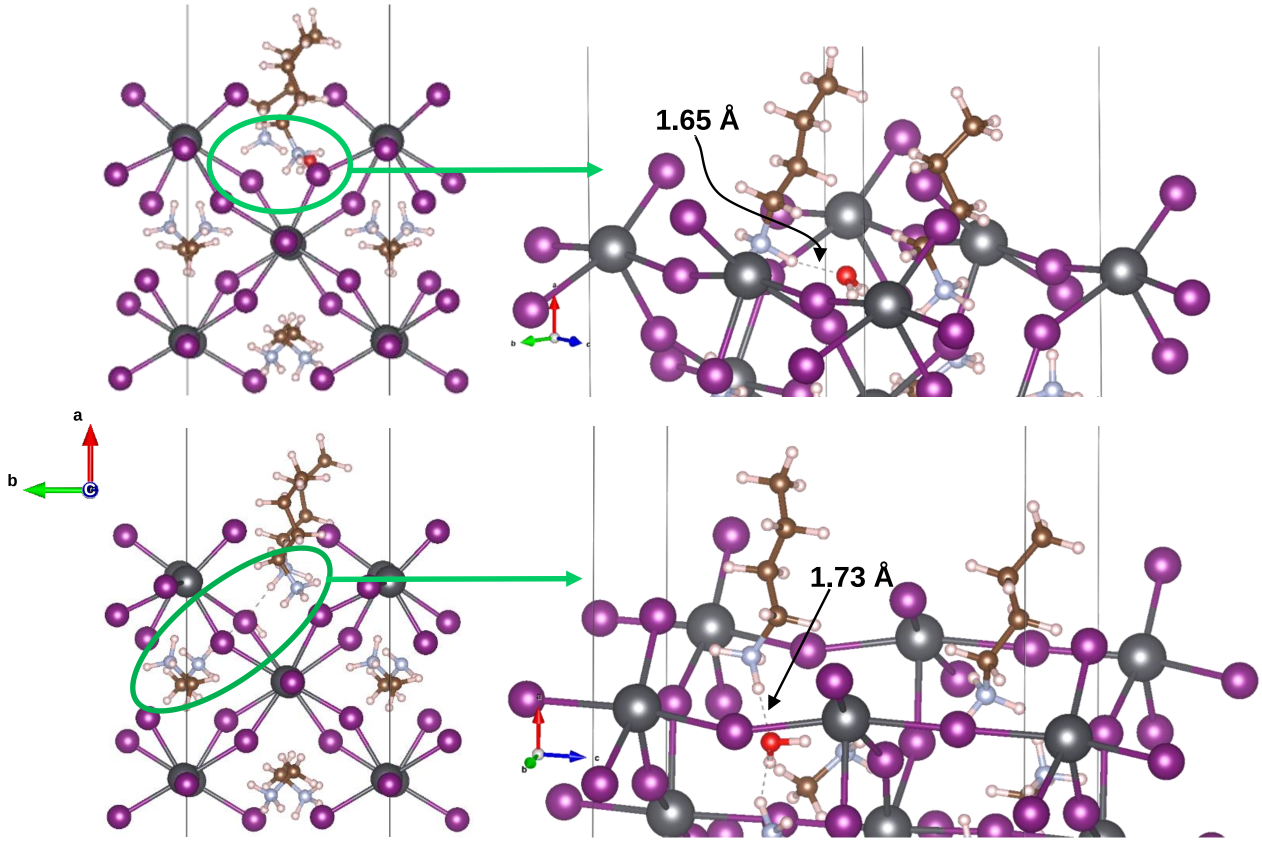

Figures S16. Representative optimized conformations of the BA-treated surfaces when water is closer to the BA.

References

[1] P.E. Blöchl, *Phys Rev B*. **1994**, *50,* 17953.

[2] J. P. Perdew, K. Burke, M. Ernzerhof, *Phys Rev Lett*. **1996**, *77,* 3865.

[3] J. Klimeš, D. R. Bowler, A. Michaelides, *J. Phys. Condens. Matter*. **2009**, *22,* 022201.

[4] W. Yang, Y. Yao, C.-Q. Wu, *J. Appl. Phys.* **2015**, *117*, 095502.

[5] D. Shi, V. Adinolfi, R. Comin, M. Yuan, E. Alarousu, A. Buin, Y. Chen, S. Hoogland, A. Rothenberger, K. Katsiev, Y. Losovyj, X. Zhang, P.A. Dowben, O. F. Mohammed, E. H. Sargent, O. M. Bakr, *Science*. **2015**, *347*, 519.

[6] I. Zarazua, S. Sidhik, T. Lopez-Luke, D. Esparza, E. De la Rosa, J. Reyes-Gomez, I. Mora-Sero, G. Garcia-Belmonte, *J. Phys. Chem. Lett.* **2017**, *8*, 6073.

[7] A. Dualeh, T. Moehl, N. Tétreault, J. Teuscher, P. Gao, M. K. Nazeeruddin, M. Grätzel, *ACS Nano*. **2014,** *8*, 362.

[8] Y. Yang, J. Song, Y. L. Zhao, L. Zhu, X. Q. Gu, Y. Q. Gu, M. Che, Y. H. Qiang, *J. Alloys Compd*. **2016**, *684*, 84.

[9] S. Yang, Y. Wang, P. Liu, Y.-B. Cheng, H. J. Zhao, H. G. Yang, *Nat. Energy*. **2016**, *1,* 15016.

[10] C. Fei, H. Wang, *Org. Electron*. **2019**, *68*, 143.

[11] G. Grancini, C. Roldan-Carmona, I. Zimmermann, E. Mosconi, X. Lee, D. Martineau, S. Narbey, F. Oswald, F. De Angelis, M. Graetzel, M. K. Nazeeruddin, *Nat. Comm*. **2017**, *8*, 15684.

[12] D. Thrithamarassery Gangadharan, Y. Han, A. Dubey, X. Gao, B. Sun, Q. Qiao, R.Izquierdo, D. Ma, *Solar RRL*. **2018**, *2*,1700215.
